# Advances, Mechanisms, and Clinical Perspectives for the In Vitro Maturation of Human Oocytes

**DOI:** 10.3390/ijms27010005

**Published:** 2025-12-19

**Authors:** Marta Gargallo-Alonso, Helen M. Picton, Clara Malo

**Affiliations:** 1 Tissue Microenvironment (TME) Lab, Aragón Institute of Engineering Research (I3A), University of Zaragoza, 50018 Zaragoza, Spain; mgargallo@unizar.es; 2 Institute for Health Research Aragón (IIS Aragón), 50009 Zaragoza, Spain; 3 Discovery and Translational Science Department, Leeds Institute of Cardiovascular and Metabolic Medicine, School of Medicine, University of Leeds, Leeds LS2 9JT, UK; h.m.picton@leeds.ac.uk

**Keywords:** in vitro maturation (IVM), oocyte, assisted reproduction, folliculogenesis, biphasic IVM, SPOM, cumulus–oocyte complex, oxidative stress, oocyte competence, PCOS, fertility preservation

## Abstract

The in vitro maturation (IVM) of human oocytes represents a valuable assisted reproductive technology that bypasses the need for full ovarian stimulation, offering safer alternatives for patients with polycystic ovary syndrome (PCOS), resistant ovary syndrome, or those requiring fertility preservation before oncological treatment. Despite its potential, IVM efficiency remains lower than that of conventional in vitro fertilization (IVF) due to incomplete understanding of the molecular and metabolic mechanisms underpinning oocyte maturation. This review summarizes recent advances in IVM, including biphasic or simulated physiological oocyte maturation (SPOM) systems, optimization of culture media through hormones, growth factors, and antioxidants, and the influence of cumulus–oocyte communication on developmental competence. We also discuss the biochemical regulation of meiosis, metabolic interactions, and gene expression patterns associated with oocyte quality. Furthermore, we examine the translational and clinical applications of IVM in human fertility treatment, highlighting its efficacy in PCOS and oncofertility cases, and the limitations that persist in replicating in vivo conditions. Emerging technologies such as microfluidic and three-dimensional culture systems show promise in enhancing oocyte competence and embryo yield. Continued research into the molecular mechanisms governing oocyte maturation will be key to improving IVM outcomes and integrating this approach as a mainstream option in reproductive medicine.

## 1. Introduction

According to the World Health Organization (WHO), approximately 48 million couples worldwide face infertility issues, representing a significant public health challenge [1]. Many turn to assisted reproductive treatments, where ovarian stimulation is a key component [2]. Controlled ovarian stimulation allows for the retrieval of multiple oocytes for in vitro fertilization (IVF) or fertility preservation, a demand growing for social or medical reasons, such as delayed motherhood or oncology treatments [3]. However, a challenge remains: around 15–20% of oocytes retrieved after stimulation do not reach maturity and are unsuitable for fertilization [4,5].

In this context, in vitro maturation (IVM) of immature oocytes has emerged as a promising approach to maximize the number of available oocytes, particularly when ovarian stimulation is unsuitable or poses risks, as in women with polycystic ovary syndrome (PCOS) [6,7]. IVM involves collecting immature oocytes and maturing them outside the body under controlled laboratory conditions before fertilization. While IVM is routinely used in domestic animal embryo production, such as cattle [8,9], its application in human assisted reproduction is still very limited [10].

The main barrier to widespread adoption of IVM in humans lies in the need to better optimize the culture conditions for human oocyte maturation, both in terms of media composition and technical manipulation [11]. Recent studies indicate that maturation and fertilization rates for IVM oocytes are variable due to differences in the developmental competence (i.e., quality) of IVM oocytes, the requirement for modification of handling techniques for harvesting compacted cumulus oocytes complexes, and to adjust laboratory conditions for oocyte culture, with 40–60% of IVM oocytes typically achieving fertilization and early cleavage division of the resulting zygotes [12,13,14]. Furthermore, implantation rates remain low, with fewer than 17% of embryos from IVM oocytes resulting in clinical pregnancies [7]. To overcome these limitations, researchers are exploring advances in optimizing culture media by adding growth factors, antioxidants, and specific nutrients that replicate the natural ovarian follicle environment [15]. Innovative techniques are being developed to modulate the oocyte microenvironment and utilize advanced biomaterials to improve oocyte maturation quality and competence [16].

A recent approach that has been proposed to improve IVM outcome is the inclusion of a pre-IVM incubation with cyclic adenosine monophosphate (cAMP) modulators to capacitate the oocyte before the induction of meiotic maturation. This method, termed Stimulated Physiological Oocyte Maturation (SPOM), also referred to as CAPA-IVM and biphasic-IVM, has garnered significant attention for its potential to enhance IVM outcomes. SPOM was initially developed in cattle and subsequently adapted for use in humans. Notably, recent clinical evidence highlights the translational application and efficacy of SPOM. Studies such as those by Albuz et al. (2010) [17] laid the foundation for SPOM protocols, while more recent reviews by Gilchrist and Smitz (2023) [18], Gilchrist et al. (2024) [19] and Navarro et al. (2024) [20] further underscore its potential utility as a means to improve oocyte competence and maturation rates. Despite these promising developments, SPOM has not been universally accepted. Some researchers remain cautious, emphasizing the need for additional evidence to substantiate its long-term effectiveness and safety. For example, Ramos Leal et al. (2022) [21] and Razza et al. (2019) [22] have raised concerns about potential variability in outcomes and the need for more standardized protocols to optimize SPOM application across diverse patient populations.

Alternatively, studies are examining the potential of co-culture systems with ovarian support cells, which may provide more physiological signals to enhance IVM outcomes [23]. These combined approaches, including SPOM and co-culture systems, aim to increase both maturation rates and successful implantation rates, offering promising solutions for couples facing infertility challenges.

## 2. Fundamentals and Regulation of Oocyte Maturation

Oocyte maturation is a fundamental process in reproduction, involving the preparation of the oocyte for fertilization through both nuclear and cytoplasmic maturation. During maturation, the oocyte resumes meiosis after remaining arrested in prophase I since primordial follicle formation and throughout all stages of follicular development to the Graafian stage. Earlier research has shown that intracellular messengers such as cyclic AMP (cAMP) in developing oocytes, together with the natriuretic peptide precursor C (NPPC) and its receptor NPR2 in surrounding granulosa cells, are crucial for sustaining meiotic arrest throughout the prolonged process of oocyte growth and maturation [24,25]. This meiotic arrest persists until the preovulatory luteinizing hormone (LH) surge triggers meiotic maturation, when fully grown oocytes in early antral and preovulatory follicles have the capacity to resume meiosis. This hormonal trigger allows the oocyte to progress to metaphase II of meiosis I, where it halts again until fertilization [26]. LH binds to the G-protein coupled LH receptor (LHR) located in the membrane of theca interna cells and the mural granulosa cells (GCs) of the ovulatory follicles. Consequently, activation of the luteinizing hormone receptor (LHR) triggers a cascade of signaling events in granulosa cells and oocytes. This activation leads to a decrease in intra-oocyte cAMP levels by downregulating the NPPC/NPR2 signaling pathway and closing the gap junctions between the oocyte and its surrounding cumulus–granulosa cells [27]. This process is essential not only for completing meiosis but also for the oocyte to acquire the necessary competence for fertilization, early embryonic development, and the accumulation of molecules and organelles vital for preimplantation development [26,28,29].

### 2.1. Nuclear Maturation

Nuclear maturation involves the oocyte’s progression through meiotic phases, advancing from prophase I to metaphase II. This process is mainly regulated by a reduction in cAMP levels, which activates maturation-promoting factor (MPF), a key cyclin-dependent kinase 1-cyclin B (CDK1-cyclin B) complex driving cell cycle progression through meiosis [29]. MPF activation induces germinal vesicle breakdown (GVBD), a critical event marking meiosis resumption triggered by the LH surge. Upon completing meiosis, the oocyte divides asymmetrically, producing a small first polar body and whilst the large oocyte retains most of the cytoplasm. The oocyte then arrests in metaphase II of meiosis I, remaining at this stage of nuclear maturation until fertilization occurs [28].

### 2.2. Cytoplasmic Maturation

In contrast, cytoplasmic maturation (or oocyte capacitation) encompasses the accumulation of molecular components and the management of the oocyte transcriptome, as well as the replication and redistribution of key organelles, chromatin condensation and organization of the structural elements needed for the oocyte to achieve fertilization, early cleavage division and embryonic development up to the activation of the embryonic genome [30]. This includes mRNA and protein synthesis, intracellular calcium storage, a burst of replication and cytoplasmic redistribution of the mitochondria, endoplasmic reticulum, cytoskeletal dynamics, cortical granule redistribution to the oocyte membrane (oolemma) to facilitate prevention of polyspermic fertilization and preparation of the oolemma for extrusion of the first polar body [26,31,32]. Microtubules and actin filaments facilitate chromosome segregation and cooperate of a number of different elements to establish the cell asymmetry necessary for polar body extrusion with minimal cytoplasm loss. Additionally, cytoplasmic maturation involves the accumulation of antioxidants, like glutathione, which protect the oocyte from oxidative during maturation and fertilization [30,31]. In this context, the subcortical maternal complex (SCMC) together with the associated cytoplasmic lattices play an essential role in the structural and functional organization of the oocyte. This complex, composed of proteins such as transducin-like enhancer of split 6 (TLE6), factor located in oocytes permitting embryonic development (FLOPED), and maternal antigen that embryos require (MATER), regulates the formation of the F-actin network, controls spindle positioning, and ensures symmetric cell division in zygotes [33]. Additionally, components such as nod-like receptor family, pyrin domain containing 4f (Nlrp4f) participate in the formation of the cytoplasmic lattice and organelle distribution [34], while nod-like receptor family, pyrin domain containing 2 (NLRP2) is involved in epigenetic reprogramming and the maintenance of postzygotic deoxyribonucleic acid (DNA) methylation [35]. Other components, such as zinc finger BED-type containing 3 (Zbed3), regulate microtubule dynamics, which are critical for female fertility and early embryonic development. In humans, homologous SCMC genes, such as nod-like receptor family, pyrin domain containing 5 (NLRP5), oocyte-expressed protein (OOEP), TLE6, and KH domain containing 3 like (KHDC3L), show specific expression in mature oocytes and early embryos, highlighting the functional conservation of this complex in mammals [36,37]. These findings underscore the importance of maternal genes and epigenetic mechanisms in the oocyte-to-embryo transition, providing a solid foundation for future research in reproductive biology and medicine.

### 2.3. Biochemical Regulation of Oocyte Maturation

Oocyte maturation is regulated by a network of coordinated biochemical signals, including endocrine, autocrine, and paracrine factors. An LH surge activates granulosa cells, reducing cAMP levels and initiating MPF pathway activation, a critical complex for meiotic resumption [28,38]. The regulation of cAMP is achieved through adenylate cyclase (AC) and phosphodiesterases (PDEs), which synthesize and degrade it, maintaining meiotic arrest until the maturation signal [39,40]. Additional pathways, such as mitogen-activated protein kinase (MAPK) and glycogen synthase kinase 3 (GSK3), regulate cell polarity and meiotic spindle formation, which are essential for correct chromosome segregation [40]. Cumulus cells also contribute vital metabolic and paracrine signals that support meiotic resumption and cytoskeletal function in the oocyte [15]. Phosphatase 2A (PP2A) signaling is another key regulator, counterbalancing MPF action to ensure proper and coordinated meiotic progression [41]. This dynamic balance between kinases and phosphatases is essential for the oocyte to complete maturation and prepare for post-fertilization embryonic development [28].

### 2.4. Molecular Regulation of Meiosis Resumption

Oocyte maturation is a complex process regulated by various molecular mechanisms, including the resumption of meiosis and interactions between oocytes and surrounding somatic cells. The resumption of meiosis in oocytes is controlled by multiple molecular factors, including regulators such as amphiregulin (AREG), epiregulin (EREG), and epidermal growth factor (EGF), along with their cognate receptors.

Resumption of Meiosis: The resumption of meiosis in oocytes is tightly regulated by cyclic nucleotides like cAMP and cGMP, which maintain meiotic arrest until the pre-ovulatory gonadotropin surge occurs, leading to a transient spike in cAMP that is crucial for oocyte development [42,43].

**Figure 1:**
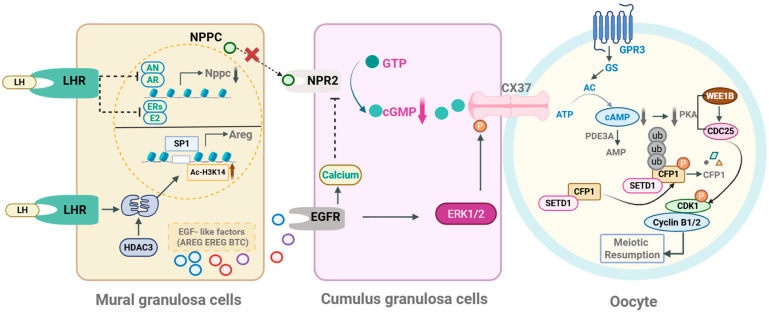
Molecular mechanisms regulating meiotic resumption in the preovulatory follicle. Created in BioRender. Gargallo-Alonso, M. (2025) https://BioRender.com/43txtpv (accessed on 7 December 2025). Figure reproduced from He et al. (2021) [48].

**Figure 2:**
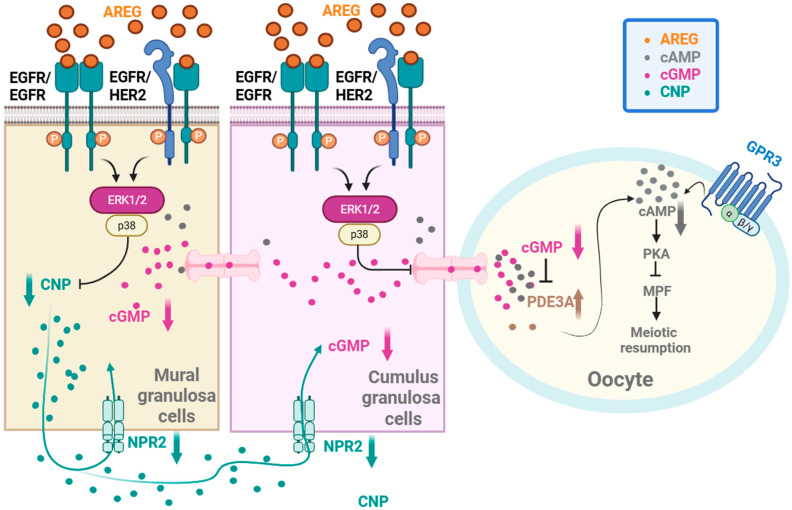
Diagram of AREG Mechanisms During Oocyte Maturation. Created in BioRender. Gargallo-Alonso, M. (2025) https://BioRender.com/0dz4z8l (accessed on 7 December 2025) Figure reproduced from Fang et al. (2023) [50].

The two way dialog between the follicular somatic cells and the presumptive gamete provide metabolic support to the oocyte from the cumulus cells and facilitate the establishment of morphogen gradients that shuttle regulatory signals between the compartments that influence preovulatory follicle development, oocyte maturation and developmental competence. The interaction between the oocyte and follicular cells is mediated through gap-junctional communication and paracrine signaling [43,44]. A pivotal event triggering meiotic resumption is the LH-induced decline in C-type natriuretic peptide (CNP), the ligand for NPR2 that sustains high cGMP levels in granulosa cells [25,27,45]. Prior to the LH surge, CNP–NPR2 signaling maintains cGMP production, keeping the oocyte in prophase I arrest. Following the LH surge, several mechanisms rapidly suppress this pathway: (i) LH downregulates NPPC (CNP) transcription in mural granulosa cells, reducing ligand availability [46]; (ii) LH promotes NPR2 dephosphorylation and inactivation, lowering its guanylyl cyclase activity and decreasing cGMP synthesis [27]; (iii) LH activates phosphodiesterases such as PDE1 and PDE5, accelerating cGMP hydrolysis [47]; and (iv) LH induces gap-junction closure between granulosa cells and the oocyte, restricting cGMP diffusion [45]. These combined actions cause a rapid fall in oocyte cGMP, relieve PDE3A inhibition and allow cAMP degradation, thereby initiating meiotic progression [48]. This LH-driven suppression of the CNP/NPR2 axis is recognized as one of the earliest and most critical triggers for meiotic resumption (Figure 1). The regulation of oocyte maturation involves multiple signaling pathways, including those mediated by follicle-stimulating hormone (FSH) and LH, which influence the levels of growth factors and peptides such as C-type natriuretic peptide (CNP), growth differentiation factor 9 (GDF9), and bone morphogenetic protein 15 (BMP15) [44,49]. In addition, a complex interaction between granulosa cell–derived EGF-like growth factors (AREG, EREG, BTC) and their receptors, together with modulators such as histone deacetylase 3 (HDAC3) and cell division cycle 25B (Cdc25b), plays an essential role in coordinating meiotic reactivation in response to hormonal signals (Figure 2). These elements work together to regulate meiotic progression and oocyte maturation, highlighting the importance of somatic–oocyte signaling pathways in this process [50].

For example, Cdc25b Phosphatase is essential for the resumption of meiosis as it activates MPF, which is necessary for cell cycle progression in oocytes [51]. In humans, granulosa cells release AREG and EREG in response to the gonadotrophins. AREG, in particular, accumulates in follicular fluid following LH stimulation, promoting oocyte maturation and cumulus expansion [42,44,49]. HDAC3 in granulosa cells acts as a negative regulator of AREG expression before the LH surge. Specifically, HDAC3 represses the expression of AREG in granulosa cells, maintaining meiotic arrest, highlighting the importance of epigenetic regulation in this process. The decrease in HDAC3 allows the transcription of AREG, promoting oocyte maturation [52]. Additionally, epigenetic modifications, such as histone acetylation and methylation, play a crucial role in the regulation of gene expression during oocyte maturation. These epigenetic changes ensure that the genes required for meiotic progression and oocyte competence are activated or repressed at the appropriate times [48].

It is important to note that although these regulatory mechanisms are broadly conserved among mammals, there are clear species-specific differences in the signaling pathways and the family members involved. For instance, AREG and EREG have been shown to be key mediators in humans and mice, while sheep may utilize different members of the EGF-like family in response to LH stimulation. For example, Cotterill et al. (2012) demonstrated that in sheep, AREG expression is significantly lower, and betacellulin (BTC) may have a more prominent role in cumulus–oocyte signaling [53]. As this review is focused on human in vitro oocyte maturation, the discussion emphasizes the signaling pathways and epigenetic regulators known to be relevant in human folliculogenesis and meiotic resumption.

### 2.5. The Metabolic Requirements and Cell–Cell Interactions Critical to Oocyte Maturation

The metabolic interplay between oocytes and their surrounding cumulus cells is essential for oocyte development and meiotic progression. Cumulus cells supply critical metabolic substrates such as glucose and pyruvate, which are metabolized to produce adenosine triphosphate (ATP) necessary for oocyte maturation [26,54,55,56]. This metabolic cooperation is facilitated by gap junctions, allowing the transfer of essential metabolites and regulatory signals that influence the meiotic state of the oocyte [57,58]. Furthermore, cumulus-mediated metabolic pathways play a pivotal role in supporting oxidative phosphorylation, ensuring sufficient energy resources for oocyte maturation [26,55,56]. The cumulus–oocyte complex (COC) is heavily dependent on glucose metabolism, which is fundamental for cumulus energy production and extracellular matrix formation, as well as being an important source of pyruvate for ATP production by the oocyte [54,55,59]. The mitochondria of all cells are the primary source of ATP, and cumulus cells play a central role in maintaining adequate ATP levels in the oocyte by providing metabolic support through gap junction communication [54,60]. Furthermore, the lipid metabolism of the oocyte, particularly through fatty acid β-oxidation, is crucial for oxidative phosphorylation, meiotic progression, and oocyte developmental competence [54,61]. Finally, cumulus cells help to safeguard oocytes from oxidative stress which is known to negatively impact oocyte quality during IVM and assisted reproductive technology (ART) procedures. Maintenance of redox balance is crucial, as physiological levels of reactive species (ROS) are needed for oocyte maturation and early embryonic development. Cumulus cells help to ensure that ROS levels remain within a beneficial range [62].

Provision of an environment that provides the correct balance of nutrients, metabolite and lipids is vital for oocyte maturation in vitro. The demands of IVM can however disrupt this delicate metabolic balance, leading to the accumulation of neutral lipids and disruption of energy homeostasis. This is reflected in the decrease in glutathione levels and ROS, as well as a lower adenosine triphosphate/adenosine diphosphate (ATP/ADP) ratio compared to in vivo matured oocytes [63,64]. To improve oocyte quality and facilitate nuclear and cytoplasmic maturation during IVM, supplementation with amino acids and other components in the culture medium can be beneficial. However, the effects of individual supplements are still a subject of debate [64]. Glucose, along with other energy substrates such as fatty acids and amino acids, plays a key role in meiotic and cytoplasmic maturation, highlighting the importance of formulating an IVM medium that adequately mimics in vivo metabolic conditions to optimize oocyte developmental competence [54,61]. This proper nutritional balance is essential for improving the outcomes of in vitro maturation and assisted reproductive technologies.

In addition to their metabolic functions, cumulus cells undergo structural changes during mucification and expansion, processes integral to oocyte maturation. These changes disrupt cumulus–oocyte contacts, reducing the supply of cAMP to the oocyte. As high cAMP levels are crucial for maintaining meiotic arrest, this reduction facilitates the resumption of meiosis [57,65]. The synthesis of hyaluronic acid during cumulus expansion is a key factor in these structural alterations, promoting successful fertilization and embryo development [66].

## 3. Evolution of Culture Conditions Used for IVM

The choice of culture media is crucial for successful IVM of human oocytes. Studies have shown that alpha-minimum essential medium (alpha-MEM) is particularly effective in supporting the growth and maturation of human oocytes, significantly improving follicular activation during the first stages of culture compared to other media such as Waymouth’s or Earle’s balanced salt solution (EBSS) [67,68]. The combination of media additives, growth factors, and metabolites added to IVM media profoundly affects maturation outcomes (see Table 1).

### 3.1. Hormones and Peptides

Follicle-stimulating hormone is commonly used in IVM culture systems to enhance maturation and reduce atresia. Supplementation of in vitro maturation medium with FSH is considered essential for supporting both nuclear and cytoplasmic maturation of human oocytes. FSH facilitates oocyte maturation, although concentrations above 0.75 IU/mL do not appear to further enhance maturation outcomes [14]. Growth hormone (GH) has also been employed to promote the maturation of human oocytes, with optimal concentrations associated with improved maturation, fertilization, and blastocyst development rates [41]. Other physiological regulators of oocyte maturation in vivo, such as the EGF-like peptide amphiregulin (AREG), may also be added to IVM media to enhance oocyte competence [26,42]. For instance, the combined use of FSH and AREG during the maturation phase has been shown to increase oocyte maturation potential and embryo yield in patients with polycystic ovary syndrome (PCOS) [69].

Insulin and insulin-like growth factor I (IGF-I) have been shown to play significant roles in the IVM of oocytes across various animal species. Insulin enhances nuclear maturation and embryonic development by promoting cumulus cell expansion and improving the cytoplasmic environment. This results in greater activity of MPF, particularly in species such as pigs [70]. IGF-I improves mitochondrial membrane potential and enhances the production of ROS in oocytes, thereby reducing apoptosis in blastocyst cells [71]. In porcine oocytes, IGF-I has been shown to improve maturation and fertilization outcomes, especially when combined with specific follicular fluids [72]. Furthermore, in bovine models, the combination of IGF-I with EGF increases mitochondrial function and glutathione levels, improving oocyte quality and developmental potential [73]. Addition of EGF has been shown to significantly enhance meiosis resumption and blastocyst development rates in ovine oocytes. However, it does not exhibit an additive effect when combined with FSH [74]. Insulin, IGF-I, and EGF appear to act synergistically or individually to support critical aspects of oocyte maturation, including cumulus cell expansion, mitochondrial activity, and apoptosis reduction. These factors, when combined with FSH, GH, or AREG, represent essential components of IVM culture systems and contribute to optimizing outcomes in assisted reproductive technologies.

### 3.2. Protein Sources

Synthetic serum substitutes [75,76]) are increasingly being used in IVM systems as a means to create a fully defined culture media as an alternative to the inclusion of human serum [77] or human serum albumen [77] or animal serum-based systems that use fetal bovine serum (FBS) or bovine serum albumen [75]. These synthetic serum substitutes offer benefits that include greater availability, cost-effectiveness, and reduced variability while eliminating risks associated with undefined biological components. Studies show that they are as effective as fetal cord serum in terms of fertilization, embryo development, and implantation rates [75]. Media supplemented with synthetic substitutes also improve embryo development and blastocyst formation [76]. Although fetal cord serum may slightly improve fertilization rates, synthetic substitutes provide a more standardized and safer option [77]. Substitutes like Knockout™ Serum Replacement (KSR) ensure a controlled environment, reducing the risks of disease transmission [75]. Overall, synthetic serum substitutes represent a reliable alternative to traditional serum supplementation, improving standardization and supporting embryo development.

### 3.3. Antioxidants and Supplementation

Antioxidants play a crucial role in maintaining the redox balance within the cellular environment, which is essential for oocyte development. Antioxidants are vital in maintaining the redox balance within the cellular environment, which is crucial for oocyte development. The cumulus cells surrounding the oocyte play a significant role in protecting it from oxidative damage by maintaining this balance. They provide essential defense mechanisms that the oocyte lacks, thereby supporting healthy embryo development [81]. Growth hormone has been shown to enhance the expression of genes associated with redox homeostasis, thereby promoting oocyte developmental competence [41], this hormone’s role in promoting the expression of these genes helps in maintaining the necessary redox balance, thereby supporting the maturation and quality of oocytes [62]. Additionally, the supplementation of culture media with lactate and super-GDF9 (an engineered form of growth differentiation factor 9) has been explored to improve COC metabolism, although these supplements alone are not sufficient to eliminate culture-induced stress [81].

Among metabolic modulators explored for improving cytoplasmic competence, L-carnitine has received particular attention due to its dual role in lipid metabolism and redox regulation. Media supplementation with L-carnitine during IVM has shown variable beneficial effects on nuclear maturation, fertilization, and embryo development across various species, including bovine, porcine, and murine models. L-carnitine is a naturally occurring quaternary amine that plays a key role in fatty acid transport into mitochondria for β-oxidation, thereby supporting cellular energy metabolism and reducing oxidative stress. In cattle, L-carnitine supplementation during IVM has been reported to enhance nuclear maturation and post-fertilization embryonic development; however, these benefits were negated after oocyte vitrification [78]. For porcine oocytes, the addition of 0.5 mg/mL of L-carnitine during IVM increased blastocyst formation following parthenogenetic activation, while reducing apoptosis and oxidative stress. It also resulted in higher intracellular glutathione (GSH) levels, indicating improved oocyte quality [79]. Similarly, in murine models, particularly those with endometriosis, L-carnitine supplementation improved oocyte quality and IVM rates by enhancing antioxidant capacity and reducing nitro-oxidative stress [80]. In contrast, other studies have reported no significant improvement in maturation or developmental outcomes following L-carnitine exposure during IVM, suggesting that its effects may depend on species, experimental conditions, and oocyte source.

Although most studies on L-carnitine supplementation during IVM have focused on its effects on oocyte quality and pre-implantation development, emerging evidence—primarily from animal research—suggests that L-carnitine may also influence pregnancy outcomes following embryo transfer. In bovine models, supplementation of IVM media with L-carnitine has been shown to improve pregnancy rates after the transfer of embryos derived from treated oocytes, indicating an enhancement of oocyte developmental competence beyond the blastocyst stage [82]. Similar findings have been reported in bovine somatic cell nuclear transfer (SCNT) embryos, where oocytes matured in the presence of L-carnitine yielded significantly higher pregnancy rates than untreated controls [83]. Other bovine studies further demonstrate improvements in oocyte maturation, mitochondrial function, and blastocyst quality, although these effects do not always translate to increased pregnancy rates, particularly when vitrification is involved [60]. Mechanistically, L-carnitine enhances mitochondrial β-oxidation, reduces oxidative stress, and supports optimal cytoskeletal and metabolic function, all of which contribute to implantation potential [61].

In humans, evidence remains limited but suggests a positive trend. One clinical trial reported that oral L-carnitine supplementation improved implantation rates, clinical pregnancy, and live birth outcomes following embryo transfer, although the embryos evaluated were not exclusively derived from IVM cycles [84]. Overall, these findings indicate that while L-carnitine shows potential to improve post-transfer pregnancy outcomes, dedicated clinical studies are still necessary to determine its true relevance within human IVM systems.

### 3.4. Oxygen Levels

Although reduced oxygen concentration during IVM is often proposed to mimic the physiological follicular niche, evidence across species does not consistently support an improvement in oocyte or embryo competence. In humans, Pham et al. (2025) [85] reported that culturing COCs under 5% O_2_ did not significantly enhance maturation outcomes compared with 20% O_2_, despite modest improvements in oocyte morphology. Anckaert et al. (2025) [86] similarly demonstrated that although 5% O_2_ influenced metabolic activity within the COC—including ATP levels and substrate utilization—it did not translate into improved embryo development. In murine models, Akin et al. (2023) [81] showed that low oxygen tension (5%) modified the bioenergetic profile of the cumulus–oocyte complex during biphasic IVM, yet these metabolic shifts did not consistently correlate with improved developmental outcomes. Other mouse studies, such as those by Banwell et al. (2007) [87], found no enhancement in implantation or fetal development under reduced oxygen concentration and even reported lower fetal and placental weights. In cattle, findings remain inconsistent: Bermejo-Alvarez et al. (2010) [88] and Pereira et al. (2010) [89] observed that 5% O_2_ can modulate gene expression or support early embryo development, but the effects are highly dependent on medium composition and metabolic substrates. Conversely, in pigs, Mingoti et al. (2009) [90] noted that higher oxygen levels (20%) may promote better blastocyst development than low oxygen conditions. Collectively, these studies indicate that oxygen reduction during IVM does not universally improve oocyte or embryo competence, and species-specific metabolic and developmental responses must be taken into account.

In summary, optimizing culture media and hormonal supplementation remains essential for improving IVM outcomes, while the role of oxygen concentration requires further investigation to clarify its species-specific and context-dependent effects. However, challenges remain in precisely recreating physiological conditions within static in vitro environments. Research continues to explore strategies to improve nuclear and cytoplasmic maturation synchronization and maximize clinical success rates.

### 3.5. Overview of Group vs. Single COC Culture

The culture of COCs can be performed either individually or in groups, and this choice can influence the outcomes of IVM. Studies have explored the effects of these different culture methods on oocyte maturation and subsequent embryological development (see Table 2). Comparative Outcomes:

Maturation Rates: The effects of single versus group cumulus–oocyte complex (COC) culture on oocyte maturation and subsequent embryo development have been widely investigated, with outcomes varying according to species, culture media, and the number of COCs used per droplet. In general, single COC culture tends to result in lower maturation and blastocyst rates compared with group culture, likely due to reduced concentrations of paracrine factors secreted by neighboring cumulus cells [8,91]. For human oocytes, Pham et al. reported no significant difference in maturation outcomes between single and group COC IVM in women with PCOS, although most studies suggest that group culture generally yields higher developmental potential [92]. Similarly, in bovine models, maturation performance and developmental potential have been shown to depend on the number of COCs cultured together, with group systems generally providing superior results [8]. These findings highlight the importance of culture density and collective signaling in optimizing IVM efficiency across species.

Embryo Development: The developmental competence of embryos derived from both single and group COC cultures appears comparable in some reports. For example, in a study involving human germinal vesicle-stage oocytes, Goud et al. (1998) [93] reported no significant differences in the number of good-quality embryos obtained between individual and group culture systems, highlighting the essential role of cumulus cells and epidermal growth factor in supporting maturation regardless of culture format [94]. However, it is generally observed across species that group cultures tend to yield better developmental outcomes than single cultures. This advantage is largely influenced by the nature of the culture system employed, which varies widely between laboratories and species, affecting the extent of paracrine signaling and microenvironmental support. Additionally, coculturing COCs with denuded oocytes (DOs) has shown synergistic effects, enhancing embryo development and quality likely through improved paracrine interactions, as demonstrated in caprine models [92].

Coculture Benefits: Coculturing COCs with other cell types, such as denuded oocytes or cumulus-derived somatic cells, can enhance maturation and developmental outcomes. This approach has been shown to improve nuclear and cytoplasmic maturation, prevent zona pellucida hardening, and increase the expression of beneficial genes in oocytes [95,96].

In humans, both single and grouped cumulus–oocyte complex (COC) cultures are viable methods for IVM, with comparable outcomes in terms of oocyte maturation and embryo quality. The choice between these approaches often depends on specific experimental conditions or laboratory preferences. Early work by Wynn et al. (1998) demonstrated that single human oocyte IVM can achieve satisfactory maturation and fertilization rates, supporting the feasibility and efficacy of this approach in clinical and research settings [92]. Moreover, co-culturing with supportive somatic cells may further enhance oocyte developmental potential, suggesting a promising strategy to optimize human IVM systems.

In other species, such as bovine and caprine models, group culture of oocytes has been shown to significantly improve meiotic competence and subsequent developmental potential compared to individual culture, under both cell-free and granulosa cell co-culture conditions [94,95]. This improvement is largely attributed to paracrine communication among oocytes, which appears absent in isolated culture—even when granulosa cell monolayers are present. Notably, the size of the oocyte group in relation to the volume of culture medium used is critically important, with optimal developmental outcomes reported in groups of 20 to 40 oocytes [94]. In mice, group culture influences both nuclear and cytoplasmic maturation, although the degree of this effect may vary depending on the species and the specific composition of the culture medium. These interspecies findings highlight the importance of paracrine and environmental factors in IVM optimization across mammals [96].

### 3.6. Gene Expression and Developmental Competence

The gene expression profiles of cumulus cells and oocytes reveal that genes involved in critical processes such as cell signaling, metabolism, and extracellular matrix formation, are indispensable for proper oocyte maturation [97,98]. However, aberrant gene expression in cumulus cells, especially under suboptimal IVM conditions, can detrimentally affect oocyte development. This highlights the necessity for carefully optimized culture environments to support normal gene expression and enhance oocyte quality [98].

In humans, studies such as that by Feuerstein et al. (2007) [99] have demonstrated that gene expression patterns in cumulus cells serve as valuable biomarkers for predicting oocyte competence. Their work identified that cumulus cells from competent oocytes display upregulated expression of genes involved in cell communication, extracellular matrix remodeling, and metabolic processes, including hyaluronan synthase 2 (HAS2), rostaglandin-endoperoxide synthase 2 (PTGS2), and gremlin 1 (GREM1), which correlate with successful fertilization and embryo development. Conversely, altered or diminished expression of these genes is associated with reduced developmental potential, emphasizing the critical role of cumulus cell gene regulation in oocyte quality and subsequent embryo viability [99].

Extensive evidence from animal models further underscores the importance of precise gene regulation in developmental competence. In cumulus cells, high expression levels of genes such as FSHR, insulin-like growth factor 1 receptor (IGF-1R), anti-müllerian hormone (AMH), and epidermal growth factor receptor (EGFR) are associated with oocytes capable of reaching the blastocyst stage, whereas elevated expression of pro-apoptotic genes like Bax and Caspase-3 correlates with reduced competence [100]. This gene expression profiling provides a non-invasive means to predict oocyte quality.

In oocytes themselves, maternal RNA and proteins accumulated during folliculogenesis are critical, but only a subset of expressed genes are directly linked to developmental competence. Notably, mitochondrial and energy metabolism-related genes (adenylate kinase 2 (AK2), enoyl-CoA hydratase short chain 1 (ECHS1), succinate-CoA ligase GDP-forming subunit alpha (SUCLG1)) play a pivotal role in oocyte quality [101,102].

Furthermore, the microenvironment and culture conditions, including embryo density and oxidative stress, influence the expression of stress and metabolic genes, thereby impacting embryonic quality and developmental potential. Suboptimal culture environments can induce aberrant gene expression profiles and reduce embryo viability [103,104].

While a significant body of literature addresses these mechanisms in humans, the large and well-established evidence base from animal IVM models provides invaluable insights into the molecular regulation of oocyte competence.

## 4. Differences Between In Vivo and In Vitro Oocyte Maturation Quality

The quality of in vivo-matured oocytes compared to in vitro-matured oocytes has significant implications for assisted reproductive technologies. Differences in developmental competence, gene expression profiles, and overall oocyte quality underscore the complexity of mimicking in vivo physiological conditions. Oocytes matured in vivo demonstrate greater developmental competence and result in higher-quality embryos compared to those matured in vitro. This is evidenced by higher rates of good-quality embryos and improved clinical pregnancy rates [105,106]. In vivo maturation contributes to a more physiologically appropriate degree of chromosomal aberrations and a higher cell count in blastocysts, indicating better embryo quality [107].

In contrast, oocytes matured in vitro exhibit significant differences in gene expression profiles. In human oocytes matured in vitro, more than 2000 genes are expressed at higher levels in the in vitro-matured oocytes, which may lead to developmental incompetence due to dysregulation in gene transcription or post-transcriptional modifications [107,108]. Co-culturing in vitro-matured human oocytes with cumulus cells—particularly those exhibiting expanded morphology, as typically seen in in vivo-matured complexes—improves their gene expression profiles, making them more comparable to in vivo-matured oocytes [107]. Moreover, the morphology of the cumulus, along with the overall culture conditions, has a significant influence on oocyte developmental competence. Depending on the species studied the presence of cumulus cells and supplementation with growth factors, such as EGF, AREG and EREG can markedly improve the maturation and developmental competence of IVM oocytes [91,109].

Clinical outcomes show that pregnancy rates are generally higher in cycles where in vivo-matured oocytes are used compared to those that rely exclusively on in vitro-matured oocytes [110,111]. This suggests that in vivo maturation results in oocytes of higher developmental competence and better clinical outcomes, consistent with transcriptomic differences observed between in vivo- and in vitro-matured oocytes [108]. At the molecular and cellular levels, the absence of specific proteins and the lack of a natural ovarian environment in in vitro conditions contribute to the reduced developmental competence of in vitro-matured oocytes. The natural LH surge and the in vivo follicular environment are crucial for proper oocyte maturation [111,112].

In summary, in vivo-matured oocytes generally exhibit greater developmental competence, different gene expression profiles, and superior embryo quality compared to in vitro-matured oocytes. The natural ovarian environment and the presence of specific growth factors and proteins during in vivo maturation play critical roles in achieving these outcomes. Despite advancements in in vitro maturation techniques, challenges remain in replicating the natural conditions necessary to achieve optimal oocyte quality.

## 5. Clinical Applications of In Vitro Oocyte Maturation and Effectiveness

The IVM of oocytes is an assisted reproductive technology particularly beneficial for patients with conditions such as polycystic ovary syndrome (PCOS), resistant ovary syndrome, and for fertility preservation in cancer patients. Historically, the efficiency of IVM has been lower compared to conventional IVF; however, recent advancements have led to improved outcomes. For instance, the introduction of biphasic IVM protocols has significantly increased maturation rates and overall efficiency [113]. Additional advances in the optimization of IVM, including approaches applied to oocytes derived from ovarian tissue, have also contributed to improved developmental potential [114]. Clinical pregnancy rates in IVM have shown encouraging results, with several studies reporting outcomes comparable to those achieved with IVF, particularly in women with PCOS [115,116].

In a clinical study using kisspeptin-54 to trigger oocyte maturation, maturation occurred in 95% of participants, and the oocyte yield—defined as the number of COCs retrieved per follicle aspirated—was approximately 1.21 per follicle, reflecting an efficient oocyte recovery rather than supraphysiological maturation rates [117]. Additionally, within the context of fertility preservation, a recent systematic review reported that approximately one-third of immature oocytes retrieved from cancer patients successfully matured through IVM [118].

In the case of PCOS, IVM offers substantial advantages over conventional IVF, particularly by reducing the risk of ovarian hyperstimulation syndrome (OHSS) associated with gonadotropin-based stimulation. Current evidence indicates that IVM provides clinical pregnancy rates that are broadly comparable to those achieved with IVF, while offering a safer profile for patients at high risk of OHSS [113,115]. In resistant ovary syndrome (ROS), IVM has also emerged as a valuable strategy: Galvão et al. (2018) [119] reported a live-birth rate of 16.7% per initiated cycle and 33.3% per patient, demonstrating that IVM can yield meaningful reproductive outcomes in this otherwise challenging population [119]. Furthermore, in the context of oncofertility, IVM plays a central role in fertility preservation, enabling the recovery and maturation of immature oocytes from ovarian tissue prior to cryopreservation, thereby safeguarding reproductive potential in patients undergoing gonadotoxic treatments [114,118,119,120].

Among the challenges affecting fertility outcomes in IVM, oocyte quality remains a primary concern. Immature oocytes obtained from patients, especially those with PCOS, often display uneven quality, which can impact maturation and fertilization rates [115,121]. Additionally, the maturation environment and culture medium are critical factors; although recent improvements have increased IVM efficiency, suboptimal conditions can still result in lower maturation rates [113,121]. Patient-specific factors, such as poor oocyte maturation, also influence IVM success. For example, patients with a history of poor oocyte maturation showed unsatisfactory outcomes with IVM, with no live births reported in a particular study [109].

## 6. Future Perspectives on In Vitro Oocyte Maturation

The future of IVM in humans looks promising with the advent of SPOM or biphasic IVM, the introduction of dynamic culture systems using microfluidics, and the incorporation of biomaterials to maintain 3D structures [121]. These innovations aim to enhance the developmental competence of oocytes, improve clinical outcomes, and provide safer and more efficient ART options for patients. Further research and clinical trials are essential to validate these approaches and integrate them into routine clinical practice. For example, studies have shown that biphasic IVM can significantly enhance oocyte maturation rates, embryo quality, and the number of vitrified embryos, especially in patients with PCOS [112,122]. Additionally, this method has shown promising results in patients with gynecological malignancies, suggesting its potential for broader clinical applications [113,123].

Dynamic culture systems based on microfluidics represent a highly promising strategy for in vitro maturation (IVM) of oocytes, as they allow precise control of the cellular microenvironment—closely mimicking the dynamic conditions of the ovarian follicle. Microfluidic devices provide continuous nutrient flow, waste removal, and controlled gradients of hormones, metabolites, and oxygen, thereby supporting both metabolic and developmental competence of oocytes. Although direct studies on human oocyte IVM using microfluidics remain limited, Vos et al. (2021) [124] reviewed the potential of microfluidic platforms to enhance IVM outcomes, particularly through improved regulation of hormone and nutrient delivery. Mastrorocco et al. (2020) [125] demonstrated that a 3D microencapsulation system for cumulus–oocyte complexes enhances maturation and cumulus expansion, recreating a physiologically relevant microenvironment. In animal models, Akin et al. (2023) [81] applied a biphasic microfluidic IVM system to mouse COCs under controlled lactate, growth factor, and low-oxygen conditions, observing improved bioenergetic profiles indicative of enhanced cytoplasmic maturation. In porcine models, Yuan et al. (2014) [126] showed that microfluidic microwell devices support individual oocyte maturation with fertilization and embryo development rates comparable to conventional IVM. Bovine studies using microfluidic sedimentation devices demonstrated that high-quality oocytes isolated under flow conditions yield higher blastocyst rates [127], and perfusion platforms in microfluidic IVM systems highlighted that flow rate influences spindle integrity, maturation, and oxidative stress [128]. Collectively, these findings suggest that microfluidic and other dynamic culture systems have the potential to improve IVM efficiency across species, and that adaptation to human IVM warrants further investigation to optimize device design, flow dynamics, and biochemical cues.

The use of biomaterials, such as alginate and collagen, to maintain a three-dimensional (3D) structure during IVM is gaining attention. These materials can encapsulate COCs in a 3D matrix, preserving their structural and functional integrity. For instance, one study demonstrated that type I collagen gel can support the structure of expanding COCs and enhance oocyte meiotic competence [129]. Similarly, alginate microbeads have been used to create a 3D culture environment, which has been shown to improve oocyte nuclear maturation, mitochondrial activity, and reduce oxidative stress [125]. While these findings suggest that 3D culture systems using biomaterials could significantly improve IVM outcomes by better mimicking in vivo conditions, other publications indicate a potential reduction in oocyte maturation rates following IVM in 3D culture. In a study investigating a millifluidic in vitro oocyte maturation system, it was found that dynamic IVM conditions, particularly when COCs were encapsulated in alginate, could lead to impaired mitochondrial distribution and activity in metaphase II oocytes compared to static conditions [130]. This suggests that while encapsulation can offer protection from shear stresses and improve bioenergetic and oxidative status, it may also negatively impact the maturation process itself. Therefore, while encapsulation can mimic physiological conditions and offer certain protective benefits, it may also hinder the overall maturation efficiency of oocytes in vitro. However, encapsulating COCs in alginates or ECM for IVM creates other disadvantages that include the need to mechanically or enzymatically release the matured oocytes from the encapsulation matrix prior to insemination.

## 7. Conclusions

In vitro maturation of oocytes has proven to be a promising tool in assisted reproduction, offering critical alternatives for patients with PCOS, poor ovarian response, and those requiring fertility preservation before oncological treatments. However, a persistent challenge remains that is to accurately replicate the complex in vivo physiological conditions necessary for optimal oocyte maturation and quality. Despite significant advancements, the efficiency of IVM still falls short compared to in vivo maturation, underscoring the need for continuous optimization of culture systems and a deeper understanding of the molecular mechanisms that influence oocyte developmental competence.

Recent developments, such as biphasic maturation—which improves maturation rates and embryo quality through a pre-maturation approach mediated by factors like CNP—represent a significant step forward. These innovations benefit not only patients with polycystic ovaries but also those with gynecological malignancies, broadening the clinical applications of IVM. Additionally, dynamic culture systems using microfluidics offer a more physiologically relevant environment, mimicking natural ovarian conditions through continuous nutrient supply and efficient waste removal.

Overall, while technological advances are redefining the potential of IVM, further research is needed to address current limitations and optimize protocols. Integrating innovative culture methods that better support oocyte biology and confer improved developmental competence could allow IVM to achieve outcomes closer to natural oocyte maturation.

Future progress in IVM will depend on the integration of molecular, technological, and clinical research. Large-scale, multicenter clinical trials are particularly essential to validate laboratory findings, establish standardized protocols, and consolidate IVM as a safe, effective, and mainstream alternative to conventional IVF.

## Figures and Tables

**Table 1:** Table of the different media additives and their impact on IVM outcomes.

Category	Additive		Reported Effect	Species	Medium Type	Reference
Hormones and Peptides	FSH		Supports nuclear maturation, positive role in oocyte cytoplasmic maturation	Human	Serum-substituted (FCS/HFF/HSA)	Yang et al. (2021) [14]
Hormones and Peptides	FSH + AREG		Increases maturation potential, higher blastocyst quality and embryo yield in PCOS patients	Human (PCOS)	Serum-based (enhanced IVM)	Sanchez et al. (2017) [69]
Hormones and Peptides	Growth Hormone (GH)		Promoted nuclear maturation, fertilization, and blastocyst rates	Human	Not specified	Li et al. (2019) [41]
Hormones and Peptides	Insulin		Enhances nuclear maturation and cumulus expansion	Porcine	Serum-free Hormone-supplemented IVG → IVM	Kim et al. (2023) [70]
Hormones and Peptides	IGF-I	IGF-1	Reduced ROS production and improved mitochondrial activity	Bovine	Serum-based	Ascari et al. (2017) [71]
		IGF-1 + Follicular fluid	Improved maturation and fertilization depending on follicle size.	Porcine	Serum-based	Oberlender et al. (2013) [72]
Hormones and Peptides	EGF	EGF + IGF-1 + Connexin 37	Enhances meiosis resumption and blastocyst development	Bovine	Serum-based	Yang et al. (2022) [73]
		EGF + IGF-I + Estradiol	Improved nuclear maturation rates in vitro.	Ovine	Serum-based	Guler et al. (2000) [74]
Protein Sources	Serum-free media		Improved fertilization and embryo culture results compared to serum.	Human	Serum-free	Holst et al. (1990) [75]
	Synthetic Serum Substitute + α-MEM		Supported extended human embryo culture to blastocyst stage	Human	Synthetic serum-based	Desai et al. (1997) [76]
	Human serum vs. Albuminar-20		Albuminar-20 provided comparable or better results than human serum.	Human	Serum-based comparison	Staessen et al. (1990) [77]
Antioxidants and Supplements	L-carnitine		Improved maturation, cryotolerance, and embryo development in bovine.	Bovine	Supplemented	Phongnimitr et al. (2013) [78]
			Enhanced oocyte maturation and parthenogenetic embryo development.	Porcine	Supplemented	Wu et al. (2011) [79]
			Improved developmental competence of oocytes from endometriosis models.	Murine	Serum-based with supplementation	Kalehoei et al. (2022) [80]
	Lactate, super-GDF9, low O_2_ tension		Improved bioenergetic profiles and developmental potential of cumulus–oocyte complexes during IVM	Murine	Serum-free (chemically defined)	Akin et al., 2023 [81]

**Table 2:** Comparison of Group vs. Single COC Culture in human IVM.

	Single COC Culture	Group COC Culture
Maturation Rate	Slightly lower maturation rates (≈61.3%)	Typically higher rates (≈64.8%)
Embryo Quality	No significant difference	No significant difference overall
Synergistic/Paracrine Effects	Limited paracrine support	Enhanced by cumulus–cumulus signaling
Gene Expression	Lower expression of competence-related genes	Improved expression profiles under coculture

## Data Availability

No new data were created or analyzed in this study. Data sharing is not applicable to this article.
